# AIMing for survival: The impact of the free and total AIM concentration in septic patients

**DOI:** 10.3389/fimmu.2025.1685119

**Published:** 2025-11-04

**Authors:** Birte Dyck, Ulrich Bosch dos Santos, Corinna Müller, Hartmuth Nowak, Tim Rahmel, Lars Palmowski, Matthias Unterberg, Alexander Wolf, Alexander von Busch, Andrea Witowski, Britta Westhus, Barbara Sitek, Katharina Rump, Christian Putensen, Stefan Felix Ehrentraut, Alexander Zarbock, Dietrich Henzler, Nina Babel, Martin Eisenacher, Katrin Marcus, Björn Ellger, Björn Koos, Michael Adamzik, Dominik Ziehe, Lars Bergmann

**Affiliations:** ^1^ Ruhr-Universität Bochum, Knappschaft Kliniken Universitätsklinikum Bochum, Klinik für Anästhesiologie, Intensivmedizin und Schmerztherapie, Zentrum für Perioperative Präzisionsmedizin, Bochum, Germany; ^2^ Biotest Aktiengesellschaft (AG), Dreieich, Germany; ^3^ AIMunity GmbH, Bremen, Germany; ^4^ Ruhr-Universität Bochum, Knappschaft Kliniken Universitätsklinikum Bochum, Klinik für Anästhesiologie, Intensivmedizin und Schmerztherapie, Zentrum für Künstliche Intelligenz, Medizininformatik und Datenwissenschaften, Bochum, Germany; ^5^ Klinik für Anästhesiologie und Operative Intensivmedizin, Universitätsklinikum Bonn, Bonn, Germany; ^6^ Klinik für Anästhesiologie, Operative Intensivmedizin und Schmerztherapie, Universitätsklinikum Münster, Münster, Germany; ^7^ Department of Anesthesiology, Surgical Intensive Care, Emergency and Pain Medicine, Ruhr-University Bochum, Klinikum Herford, Herford, Germany; ^8^ Ruhr-University Bochum, Marien Hospital Herne, Medical Clinic I, Center for Translational Medicine, Herne, Germany; ^9^ Ruhr-University Bochum, Medizinisches Proteom-Center, Bochum, Germany; ^10^ Ruhr University Bochum, Center for Proteindiagnostics (PRODI), Medical Proteome Analysis, Bochum, Germany; ^11^ Ruhr University Bochum, Medical Faculty, CUBiMed.RUB, Core Unit Bioinformatics, Bochum, Germany; ^12^ Klinik für Anästhesiologie, Intensivmedizin und Schmerztherapie, Klinikum Westfalen, Dortmund, Germany

**Keywords:** apoptosis inhibitor of macrophages (AIM), CD5L, SepsisDataNet.NRW, IgM, Pentaglobin^®^, sepsis, biomarker, 30-day survival

## Abstract

**Background:**

Sepsis, a life-threatening condition caused by a dysregulated host response to infection, remains a major cause of mortality worldwide. Identifying reliable biomarkers for prognosis and treatment is urgently needed. This study investigates the role of the Apoptosis Inhibitor of Macrophages (AIM), also known as CD5L, as a potential prognostic biomarker and therapeutic target in sepsis.

**Methods:**

We measured free and total AIM concentrations in 90 septic patients enrolled in SepsisDataNet.NRW cohort (German Clinical Trial Registry No. DRKS00018871; http://www.sepsisdatanet.nrw). Blood samples were collected on days 1, 4, and 8, and AIM levels were quantified using ELISA. Kaplan-Meier analysis and Cox regression were performed to assess the association between AIM levels and 30-day survival. Western blot analysis was performed to detect AIM in human serum IgM and in the IgM-enriched intravenous immunoglobulin IVIG preparation Pentaglobin^®^.

**Results:**

High total AIM concentrations (>85 ng/ml) were significantly associated with improved 30-day survival on day 1 (HR: 3.131, 95% CI: 1.629-6.019, p = 0.009), 4 (HR: 2.525, 95% CI: 1.198-5.322, p = 0.0042), and day 8 (HR: 2.317, 95% CI: 0.8565-6.266, p = 0.0457). Free AIM showed a significant association with survival only on day 8 (HR: 2.374, 95% CI: 0.8721-6.461, p = 0.0393).

**Conclusion:**

Total AIM concentration is a significant predictor of a 30-day survival in sepsis, supporting its potential use as a prognostic biomarker. Our findings also suggest that AIM may serve as a valuable prognostic biomarker and a potential target for immune-modulating therapies, including IgM-enriched intravenous immunoglobulins (IVIGs).

## 1 Introduction

Sepsis, defined as a life-threatening condition by organ dysfunction caused by a dysregulated host response to infection, remains a major global health challenge (1). Despite recent medical advances, sepsis continues to claim millions of lives each year, affecting approximately 48.9 million cases and contributes to 20% of all global deaths (2).

Current therapeutic strategies for sepsis are often limited by the absence of precise and reliable biomarkers to guide early diagnosis, prediction, and therapeutic decision-making. Sepsis is highly heterogeneous, without established specific timepoints or predictable progression, making it difficult to determine the most effective therapeutic windows. Therefore, identifying biomarkers that can guide personalized treatment strategies beyond the initial onset of sepsis is critically important (3, 4).

A promising approach in this context for biomarkers and therapeutic targets is the Apoptosis Inhibitor of Macrophages (AIM), also referred as Cluster of Differentiation 5-like antigen (CD5L). AIM is a 37–40 kDa protein belonging to the scavenger receptor cysteine-rich (SRCR) superfamily (5). Secreted predominantly by tissue-specific macrophages, AIM plays a pivotal role in the innate immune system by promoting macrophages survival through the inhibition of apoptosis during infection (6). AIM’s structure is characterized by three SRCR domains rich in cysteines, which form disulfide bridges that are critical for its interactions with other molecules.

One of the most significant interactions of AIM is with Immunoglobulin M (IgM), a key player in the immune system (7). AIM circulates in the bloodstream predominantly bound to IgM, a mechanism that prevents its renal excretion and stabilizes its serum concentration (8). The biological relevant pentameric form of IgM contains a joining chain (J-chain) that creates a structural pocket accommodating AIM (8, 9). The ratio between hexameric and pentameric IgM is depending on immune stimulating agents and can shift in certain diseases (10). The molecular interaction between pentameric IgM and AIM is believed to be crucial for the biological activity and persistence of AIM in the circulation.

Beyond its anti-apoptotic function, AIM influences macrophage polarization. Macrophages can differentiate into two phenotypes: pro-inflammatory (M1) and anti-inflammatory (M2), depending on microenvironmental cues (11). AIM promotes the transition from M1 to M2 macrophages through the upregulation of the DNA-binding protein inhibitor ID3 via autophagy-dependent mechanisms (11), potentially modulating the immune response during the hyperinflammatory and immunosuppressive phases of sepsis (8).

Although AIM has been implicated in a variety of inflammatory and metabolic disorders, clinical data regarding its role as a prognostic biomarker in sepsis are limited. In this study, we measured both free and total serum AIM levels in septic patients and analyzed their relationship with 30-day mortality. We hypothesized that higher serum AIM concentrations are associated with improved outcomes in sepsis.

## 2 Study design and methods

### 2.1 Study design and cohort

this study utilized biospecimens and clinical data from the SepsisDataNet.NRW cohort (German Clinical Trial Registry No. DRKS00018871; http://www.sepsisdatanet.nrw). Patients were enrolled based on fulfillment of the Sepsis-3 criteria. The study protocol was approved by the Ethics Committee of the Medical Faculty of Ruhr-University Bochum (protocol no. 18-6606-BR/5047-14). Recruitment took place between 1st of March 2018 until 28st of February of 2020 across seven intensive care units (ICU) in tertiary care and university hospitals in the German state of North Rhine-Westphalia. Written informed consent was obtained from all participants or their legal representatives.

Inclusion criteria for the SepsisDataNet.NRW were as follows:

- Fulfillment of SEPSIS-3 criteria.
- Age ≥18 years at the time of ICU admission.
- Availability of informed consent.

### 2.2 Clinical data and patient characteristics

Clinical and demographic data, including vitals, laboratory parameters, point-of-care-diagnostics, and length of ICU-stay were captured using the CentraXX software platform (Kairos GmbH, Bochum, Germany). All data were pseudonymized in accordance with ethical and data protection guidelines. Missing data was supplemented through a retrospective review of the patients’ medical by experienced physicians. Where applicable, clinical data within ±12 h of sepsis onset were included. Sequential Organ Failure Assessment (SOFA) scores were manually calculated at each site by an experienced physician. All patients received care according to the current international guidelines for sepsis management. The final study cohort compromised 90 patients. Baseline characteristics are summarized in **Table 1**.

**Table 1:** Baseline characteristics of the patient cohort.

Characteristics	Value
n	90
Male n (%)	37 (41%)
Age Median [IQR]	64 [53-72]
Admission SOFA Score, median [IQR]	9 [6-12]
ICU LOS, days, median [IQR]	9 [4-16]
30-day survival	54 (60%)
Infection focus	n (%)
Lung	28 (31%)
Abdomen	60 (67%)
Urogenital	1 (1%)
Cardiovascular	1 (1%)

IQR, interquartile range; ICU, intensive care unit; LOS, length of stay.

### 2.3 Procedures

#### 2.3.1 Sample collection and processing

Peripheral venous blood was collected in 9.0 mL Serum CAT tubes (Sarstedt, Nümbrecht, Germany) and centrifuged for 4 min at 4000 x g. The serum supernatant was aliquoted and stored at -80 °C until further analysis.

#### 2.3.2 Determination of AIM-concentration via ELISA

Quantification of total AIM (comprising both IgM-bound AIM and free AIM) was performed using the *Human CD5L (CD5 Antigen-like) ELISA Kit* (AssayGenie, Dublin, Ireland; Cat. No. HUES03547). This sandwich ELISA (96-well format) has a reported detection range of 0.78–50 ng/mL and a sensitivity (limit of detection) of 0.47 ng/mL, with a required sample volume of 100 µL. According to the manufacturer, intra- and inter-assay coefficients of variation are both below 10%. The assay captures both free circulating AIM and AIM bound to IgM. Serum samples from day 1, 4 and 8 after study inclusion were analyzed. According to manufacturer’s protocol, samples were diluted 1:20, and 100 µL of each sample and respective controls were applied into microtiter test wells and incubated for 90 min at 37°C in a humified chamber. After discarding unbound substances, 100 µl of biotinylated detection antibody working solution was added and incubated for 1 hour at 37°C. Subsequently, wells were washed five times with 350 µl of wash buffer for 1–2 minutes. Then, 100 µl of HRP-conjugated working solution was added, incubated for 30 min at 37°C, followed by another five wash cycles. After the addition of the Substrate Reagent solution, incubation was carried out for 15 min at 37°C. The reaction was terminated with 50 µl of stop solution. The optical densities (OD) were determined using a microplate reader (CLARIOstar^PLUS^, BMG LABTECH, Germany). Data analysis was performed using the CLARIOstar^PLUS^ MARS software. An average over duplicates was calculated based on blank-corrected values, and a 4-parameter fit was applied. A 4-parameter logistic curve was plotted on log-log graph paper. Total AIM concentrations were calculated considering the dilution factor.

The quantification of free AIM was carried out with the *CircuLex Human AIM/CD5L/Spα ELISA Kit* (MBL International, Japan; Cat. No. CY-8079) according to the manufacturer’s instructions. This assay specifically detects unbound AIM and does not cross-react with IgM-bound protein. The measurement range of the kit is 1.57–100 ng/mL, with a sensitivity of 0.745 ng/mL. For the measurement, 100 µl of the diluted samples (1:20), the prepared standard solutions and respective controls were applied in duplicate on the pre-coated plate. Plates were sealed and incubated for 60 min at room temperature (RT), on an orbital microplate shaker set at 300 rpm. After incubation, the solution was decanted, and the plate washed four times with 300 µl washing buffer for 1 min. Subsequently, 100 µl of HRP- conjugated detection antibody solution was dispensed into each well, and the plate was again sealed and incubated for 60 min at RT under shaking. Following another wash cycle, 100 µl of the Substrate Reagent added. The plate was protected from light using aluminum foil and incubated for 20 min at RT under shaking. Finally, the Stop Solution was added in the same order as the Substrate Reagent and the absorbance was measured at 450 nm using a microplate reader (BMG Labtech, Germany). Data analysis was conducted analogously to the ELISA measurement of total AIM.

#### 2.3.3 Statistical analysis

To evaluate the association between AIM concentration on 30-day survival, Kaplan-Meier analyses and multivariate Cox regression were performed. First, a cut-off value for AIM concentration was determined by receiver operating characteristic (ROC) curve analysis using the Youden index for each individual time point. Patients were stratified into two groups based on this threshold: those with elevated AIM levels (above the cut-off) and those with reduced AIM levels (below the cut-off) followed by Kaplan-Meier analyses. Subsequently, a multivariate survival analysis was performed using Cox regression. All analyses were performed using SPSS software Version 29 (IBM, USA). Graphical visualizations were generated using GraphPad Prism (version 8.0; GraphPad Software, USA).

## 3 Results

### 3.1 Patient characteristics

We included 90 patients fulfilling Sepsis-3 criteria from four ICUs in our study. The cohort consisted of 37 male patients (46%) with a mean age of 64 (± 12) years. The median SOFA score at the time of inclusion was 9 (IQR: 6–12), and the 30-day survival rate was 60%. Further details on baseline characteristics are presented in **Table 1**.

### 3.2 Prognostic impact of free AIM on 30-day survival

**Figure 1:**
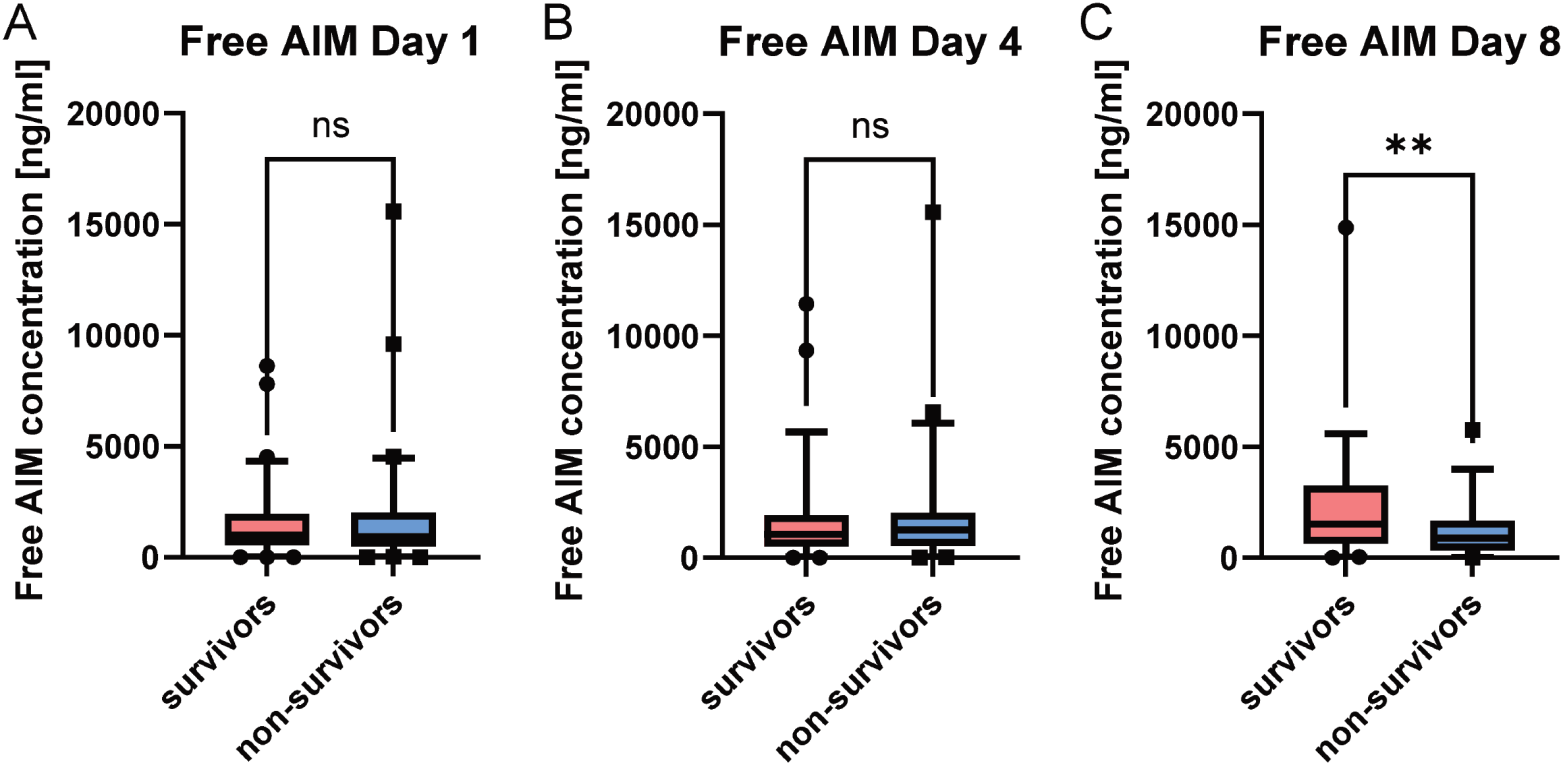
Free AIM concentrations in sepsis patients stratified by survival status. Boxplots depict free AIM concentrations on day 1 **(A)** (n = 90), day 4 **(B)** (n = 74), and day 8 **(C)** (n=54) after study inclusion, comparing survivors (red) and non-survivors (blue). Statistical testing was performed using the Mann–Whitney U test. No significant differences were observed on day 1 or day 4, whereas survivors displayed significantly higher free AIM concentrations on day 8 (p = 0.0062). Boxes represent the 5th–95th percentile range, horizontal lines indicate the median. ** p < 0.01.

**Figure 2:**
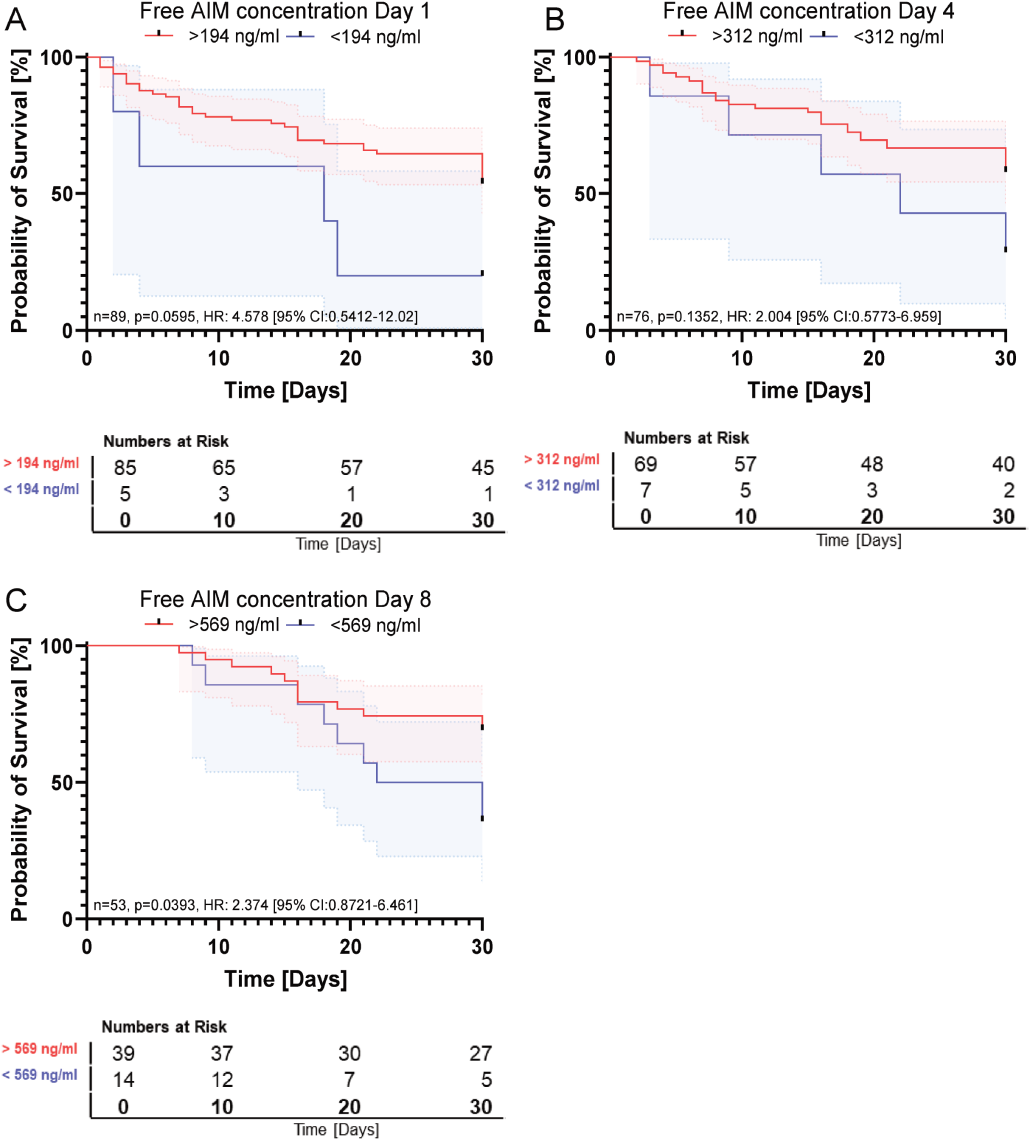
Association between free AIM concentration on survival. **(A)** Free AIM concentration on day 1. Kaplan-Meyer Analysis: n=89, p=0.0595, HR: 4.578 [95% CI:0.5412-12.02]. **(B)** Free AIM concentration on day 4. Kaplan-Meyer Analysis: n=76, p=0.1352, HR: 2.004 [95% CI:0.5773-6.959] **(C)** Free AIM concentration on day 8. Kaplan-Meyer Analysis: n=53, p=0.0393, HR: 2.374 [95% CI:0.8721-6.461].

Direct group comparison revealed that free AIM concentrations were significantly higher in survivors than in non-survivors at day 8 (p = 0.0062), while no significant differences were observed at earlier time points (**Figure 1**). Although Kaplan-Meier analysis suggested a potential trend toward improved survival with higher free AIM concentrations on day 1, multivariate Cox regression analysis, adjusted for gender, age, and SOFA score at sepsis admission, did not confirm a significant protective effect (**Table 2**, HR: 2.46 [95% CL: 0.70-8.61], p = 0.161). The optimal cutoff values for free AIM concentrations were derived from Receiver Operating Characteristic (ROC) curve analysis, with the corresponding area under the curve (AUC) values presented in **Supplementary Figure 2**. However, a significant survival benefit was observed for patients with elevated free AIM concentration on day 8 post-inclusion (**Figure 2c**, p = 0.0393, HR: 2.374 [95% CI: 0.8721–6.461]).

**Table 2:** Multivariate Cox regression analysis assessing the association of total and free AIM concentration with 30-day survival, adjusted for age, gender and SOFA Score.

	Total AIM	Free AIM
AIM-concentration	HR: 2.79 [95% CL: 1.08-7.12] p = 0.034	HR: 2.46 [95% CL: 0.70-8.61] p = 0.161
Age	HR: 1.020 [95% CL:0.997-1.042] p = 0.083	HR: 1.024 [95% CL: 1.001-1.048] p = 0.044
Admission SOFA Score	HR: 1.155 [95% CL:1.060-1.259] p < 0.001	HR: 1.184[95% CL: 1.081-1.296] p < 0.001
Gender	HR: 1.145 [95% CL:0.615-2.132] p = 0.670	HR: 0.974 [95% CL: 0.495-1.914] p = 0.939

### 3.3 Prognostic impact of total AIM on 30-day survival

**Figure 3:**
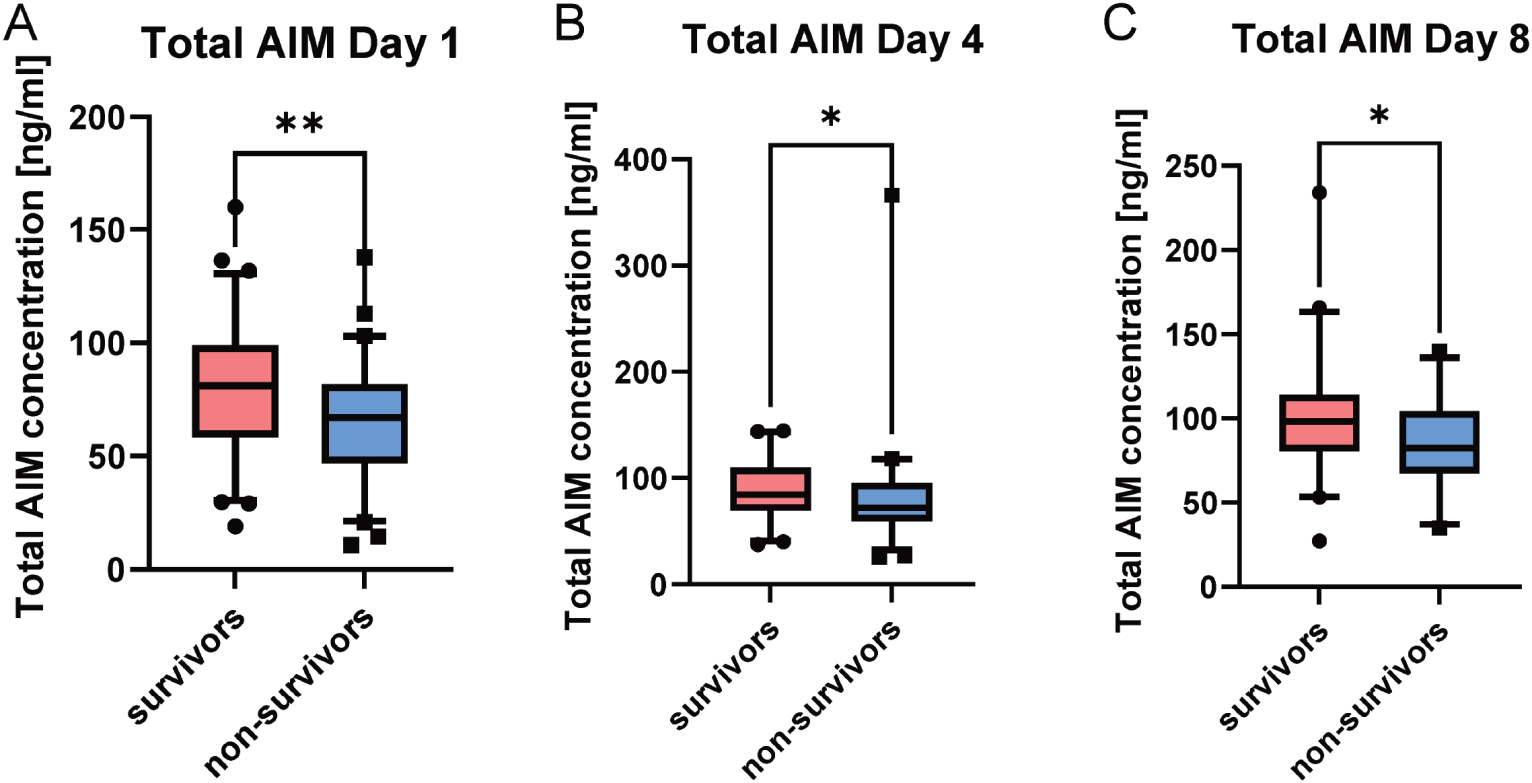
Total AIM concentrations in sepsis patients stratified by survival status. Boxplots depict total AIM concentrations on day 1 **(A)** (n = 90), day 4 **(B)** (n = 74), and day 8 **(C)** (n=54) after study inclusion, comparing survivors (red) and non-survivors (blue). Statistical analysis was performed using the Mann–Whitney U test. Survivors exhibited significantly higher total AIM concentrations at all three time points (day 1 p = 0.014, day 4 p = 0,0247 and day 8 p = 0.0199). Boxes represent the 5th–95th percentile range, horizontal lines indicate the median. * p<0.05; ** p<0.01.

**Figure 4:**
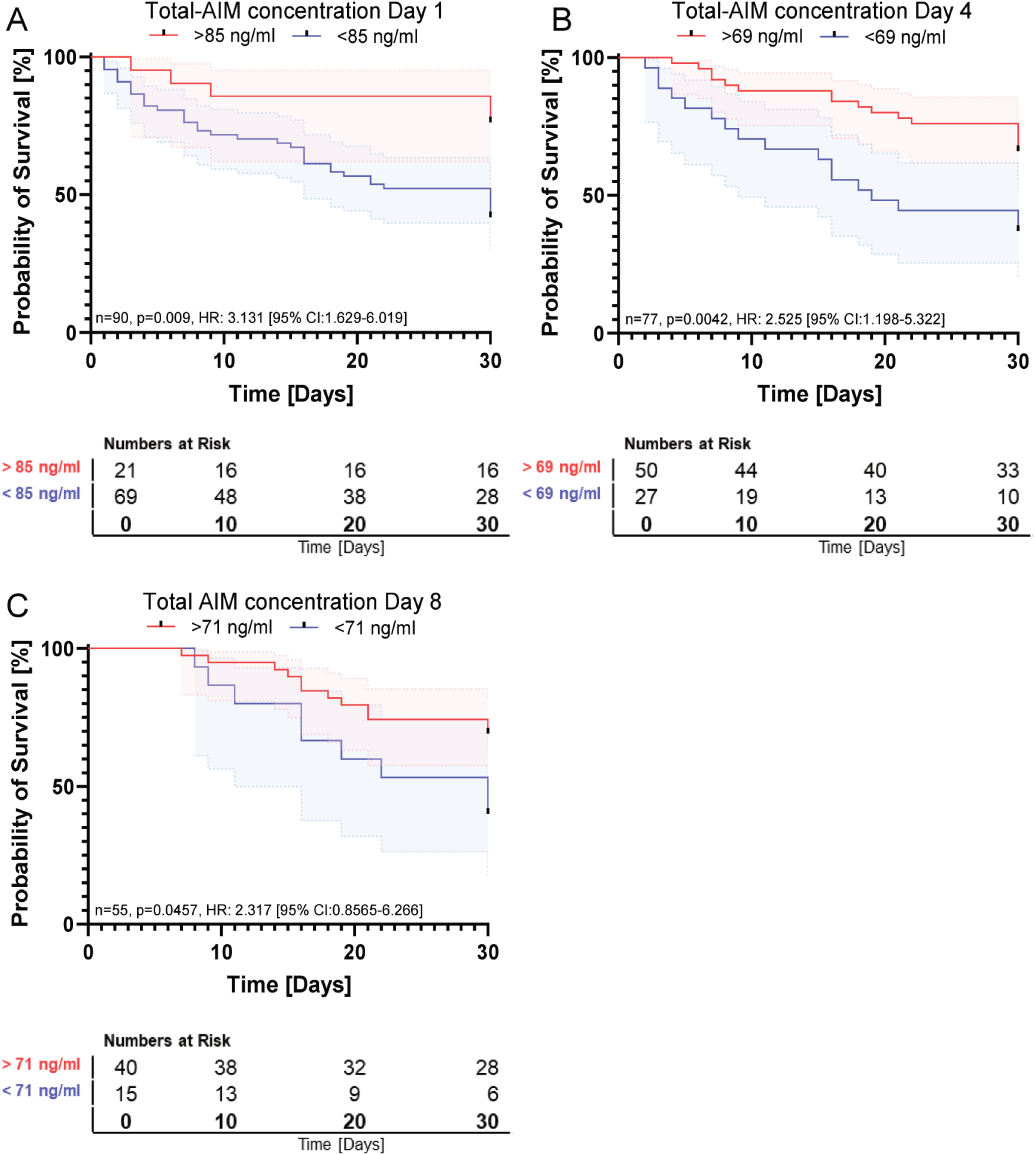
Association between total AIM concentration and survival. **(A)** Total-AIM concentration on day 1. Kaplan-Meyer Analysis: n=90, p=0.009, HR: 3.131 [95% CI:1.629-6.019]. **(B)** Total-AIM concentration on day 4. Kaplan-Meyer Analysis: n=77, p=0.0042, HR: 2.525 [95% CI:1.198-5.322]. **(C)** Total-AIM concentration on day 8. Kaplan-Meyer Analysis: n=55, p=0.0457, HR: 2.317 [95% CI:0.8565-6.266].

Boxplot analysis using the Mann-Whitney U test demonstrated significantly higher total AIM concentrations in survivors compared to non-survivors on day 1 (p = 0.014), day 4 (p = 0,0247), and day 8 (p = 0.0199) (see **Figure 3**). Kaplan-Meier analysis (**Figure 4**) revealed a significant association between higher total AIM levels and improved 30-day survival on day 1 (**Figure 4a**, p = 0.009, HR: 3.131 [95% CI: 1.629-6.019]), day 4 (**Figure 4b**, p = 0.0042, HR: 2.525 [95% CI: 1.198-5.322]), and day 8 (**Figure 4c**, p = 0.0457, HR: 2.317 [95% CI: 0.8565-6.266]). The optimal cutoff values for total AIM levels were derived from Receiver Operating Characteristic (ROC) curve analysis, with corresponding area under the curve (AUC) values, which are presented in **Supplementary Figure 3**. Further, multivariate Cox regression analysis confirmed that elevated total AIM levels on day 1 were independently associated with improved survival (HR: 2.79 [95% CL: 1.08-7.12], p = 0.034), even after adjusting for age, gender and SOFA Score (**Table 2**). In contrast, age (HR: 1.020 [95% CL:0.997-1.042], p = 0.083) and gender (HR: 1.145 [95% CL:0.615-2.132], p = 0.670) were not significant predictors. The SOFA score at admission remained a strong independent predictor of mortality (HR: 1.155 [95% CL:1.060-1.259], p < 0.001).

## 4 Discussion

Our study provides valuable insights into the role of AIM (apoptosis inhibitor of macrophages) as both a prognostic marker and a potential therapeutic target in critically ill patients with sepsis. Despite significant advances in intensive care medicine, sepsis remains a major global health burden with persistently high mortality rates. A major obstacle to improved patient outcomes is the absence of reliable biomarkers capable of predicting disease progression or guiding individualized therapeutic strategies.

Our results demonstrate that total AIM concentration is a robust and consistent predictor of 30-day survival. Kaplan-Meier analyses revealed significant associations between higher total AIM concentration and improved survival at all measured time points (days 1, 4, and 8). These findings suggest that elevated circulating AIM levels correlate with better clinical outcomes. This was mirrored by direct group comparisons, where survivors consistently displayed higher total AIM concentrations than non-survivors (**Figure 3**). Notably, sustained high total AIM concentrations over the initial 8-day period were associated with improved survival, offering potential for early risk stratification and timely intervention even beyond the initial onset of sepsis. As total AIM is stabilized by binding to IgM (12), it may more reliably reflect systemic availability and immune modulation over time. This stability could also facilitate its use in centralized biomarker assessment, making AIM quantification accessible even for smaller healthcare facilities and enhancing clinical decision-making across diverse settings.

Although patients with higher free AIM concentrations on day 1 showed a trend toward improved 30-day survival (p = 0.0595), statistical significance was not reached until day 8. At this time point, survivors displayed significantly higher free AIM concentrations compared to non-survivors (**Figure 1**). This temporal shift suggests that the prognostic relevance of the unbound fraction emerges only after early compensatory mechanisms have subsided or organ injury has progressed. Given that free AIM is subject to rapid renal clearance, its plasma levels likely reflect dynamic pathophysiological processes rather than stable immunological status (13).

To further assess the independent contribution of AIM to patient outcome, multivariate Cox regression analyses were performed including AIM concentrations at all measured time points together with age and cardiovascular comorbidities (see **Supplementary Tables 1** and **2**). These analyses identified free AIM on day 8 [HR: 0.999 (0.999–1.000); p = 0.007], cardiovascular comorbidity [HR: 0.242 (0.083–0.702); p = 0.009], and age [HR: 1.065 (1.021–1.110); p = 0.003] as independent factors associated with 30-day survival. Similarly, total AIM on day 1 [HR: 0.974 (0.955–0.993); p = 0.008], together with cardiovascular comorbidity [HR: 0.205 (0.070–0.598); p = 0.004] and age [HR: 1.044 (1.004–1.086); p = 0.031], emerged as independent predictors of outcome.

These results suggest that total AIM provides early prognostic information, whereas free AIM becomes relevant at later disease stages. Both parameters complement established clinical variables and likely capture different aspects of the host response, with total AIM reflecting the stable, IgM-bound pool and free AIM representing the dynamic, unbound fraction that responds to ongoing immune activation and tissue injury. Together, they emphasize that AIM adds contextual information to traditional clinical markers rather than acting as a single, dominant determinant of outcome.

In this regard, our longitudinal data differ from findings reported by Gao et al. (2019), who observed markedly elevated serum AIM levels at ICU admission correlating with higher SOFA scores and increased 28-day mortality (14). The apparent discrepancy between their early association and our delayed pattern may result from methodological differences (sample processing, cohort characteristics) or from the fact that Gao et al. assessed only a single baseline measurement, whereas we examined both free and total AIM longitudinally.

These divergent results highlight the importance of distinguishing between total and free AIM when assessing its prognostic relevance. Preclinical studies suggest that free AIM may exert protective in immune dysregulation (15). However, the therapeutic utility of the free form of AIM may be limited by its short persistence in circulation. In contrast, binding to IgM markedly prolongs its half-life by preventing renal loss and stabilizing AIM within the plasma compartment (16), potentially enabling sustained therapeutic effects.

Since IgM is a key component of IgM-enriched intravenous immunoglobulins (IVIGs) formulations such as Pentaglobin^®^, whose clinical efficacy in sepsis remains under debate (17), we explored whether these preparations contain AIM (see **Supplementary Data Sheet 1**). Western blot analysis confirmed the presence of AIM in Pentaglobin^®^ raising the possibility that IVIGs may serve as a new therapeutic application for patients with deficient AIM levels. Nevertheless, quantitative approaches such as ELISA or mass spectrometry should be applied in future work to evaluate therapeutic AIM content more precisely.

In summary, our findings identify total AIM as a promising prognostic biomarker in sepsis, associated with improved survival across multiple time points. While free AIM becomes relevant only at later disease stages, this delayed association may limit its practical utility in the dynamic clinical setting of sepsis.

Importantly, no single biomarker can capture the full complexity of sepsis. As emphasized in recent calls for precision medicine in sepsis (Giamarellos-Bourboulis et. al, 2024), patient stratification will require integration of multiple biomarker layers (18). Within this framework, AIM may represent one useful dimension that complements established predictors. Beyond its value as a prognostic marker, AIM might also open avenues for therapeutic exploration, for instance in combination with IgM-enriched immunoglobulin preparations or other immune-modulating agents. Further studies are needed to clarify these roles and to evaluate AIM within multimodal strategies for sepsis management.

### 4.1 Limitations

It is important to acknowledge the limitations of this study. Although prospectively enrolled and based on high-quality data, the cohort size was relatively small, which may limit the generalizability of our findings. Furthermore, threshold values for total AIM and free AIM differed across time points, underscoring the need for larger validation cohorts to establish standardized and clinical meaningful cutoff values. As part of this next step, we aim to extend our analyses to larger patient populations and to include critically ill non-septic controls. Such comparative cohorts will allow us to better delineate the specificity of AIM.

## 5 Conclusion

Our study identifies total AIM as a novel and clinically relevant prognostic biomarker in sepsis, independently associated with improved 30-day survival. The consistent association across multiple time points and robustness in multivariate analysis supports its potential utility for early risk stratification and longitudinal patient monitoring. While free AIM demonstrated some temporal association with outcome, particularly total AIM, likely reflecting a more stable and bioavailable form, emerged as the more reliable predictor. These findings offer a promising avenue for biomarker-guided management of sepsis and suggest AIM as a candidate for future therapeutic exploration. However, prospective validation in larger, independent cohorts is essential.

## Data Availability

The original contributions presented in the study are included in the article/**Supplementary Material**. Further inquiries can be directed to the corresponding author.
