# Supplementary material for: AIMing for survival: The impact of the free and total AIM concentration in septic patients
Source: Front Immunol. 2025 Nov 4;16:1685119. doi: 10.3389/fimmu.2025.1685119 (PMC12623382; doi:10.3389/fimmu.2025.1685119)
Supplement: Supplementary file 1 [file DataSheet1.docx]

92AIMing for Survival: The Impact of the free and total AIM Concentration in Septic Patients

**Birte Dyck#^1^, Ulrich Bosch dos Santos#^2,3^, Corinna Müller^2^, Hartmuth Nowak^1,11^, Tim Rahmel^1^, Lars Palmowski^1^, Matthias Unterberg^1^, Alexander Wolf^1^, Alexander von Busch^1^, Andrea Witowski^1^, Britta Westhus^1^, Barbara Sitek^1^, Katharina Rump^1^, Christian Putensen^4^, Stefan Felix Ehrentraut^4^, Alexander Zarbock^5^, Dietrich Henzler^6^, Nina Babel^7^, Martin Eisenacher^8,9^, Katrin Marcus^8^, Björn Ellger^10^, Björn Koos^1^, Michael Adamzik^1^, Dominik Ziehe^§*1^, Lars Bergmann^§1^**

^1^ Zentrum für perioperative Präzisionsmedizin der Klinik für Anästhesiologie, Intensivmedizin und Schmerztherapie, Knappschaft Kliniken Universitätsklinikum Bochum, Bochum, Germany

^2^ Biotest AG, Dreieich, Germany

^3^ AIMunity GmbH, Bremen, Germany

^4^ Klinik für Anästhesiologie und Operative Intensivmedizin, Universitätsklinikum Bonn, Bonn, Germany

^5^ Klinik für Anästhesiologie, Operative Intensivmedizin und Schmerztherapie, Universitätsklinikum Münster, Münster, Germany

^6^ Department of Anesthesiology, Surgical Intensive Care, Emergency and Pain Medicine, Ruhr-University Bochum, Klinikum Herford, Herford, Germany

^7^ Center for Translational Medicine, Medical Clinic I, Marien Hospital Herne, University Hospital of the Ruhr-University Bochum, Herne, Germany

^8^ Medizinisches Proteom-Center, Ruhr-University Bochum, Bochum, Germany; Center for Proteindiagnostics (PRODI), Medical Proteome Analysis, Ruhr University Bochum, Bochum, Germany

^9^ CUBiMed.RUB, Core Unit Bioinformatics, Medical Faculty, Ruhr University Bochum, Bochum, Germany

^10^ Klinik für Anästhesiologie, Intensivmedizin und Schmerztherapie, Klinikum Westfalen, Dortmund, Germany

^11^Zentrum für Künstliche Intelligenz, Medizininformatik und Datenwissenschaften, Bochum, Germany

# BD and UBdS contributed equally to the manuscript

§ DZ and LB contributed equally to the manuscript

*** Correspondence:**Dr. rer. nat. Dominik Ziehe

Dominik.Ziehe@ruhr-uni-bochum.de

# Supplementary Data

**Methods**

**Western Blot**

Western blotting following SDS-PAGE was performed with Any kD™ Mini-PROTEAN^®^ TGX™ Precast Protein Gels (Bio-Rad) at 200 V for 40 min. Protein transfer was completed using Trans-Blot Turbo Mini 0.2 µm Nitrocellulose Transfer Packs (Bio-Rad) at 2.5 A and 25 V for 3 min. After two 5-min TBST washes, the membrane was blocked for 1h at RT with 5% BSA (Carl Roth) in TBST. Primary antibody incubation (IgM: Goat anti-Human IgM Secondary Antibody, #31415, HRP (Invitrogen), 1:10,000 in TBST; AIM: Anti-CD5L/CT-2 antibody #ab45406 (abcam), 1:1000 in 5% BSA in TBST) was carried out overnight at 4°C, followed by three washes with TBST. Secondary antibody incubation (Goat anti-rabbit IgG (H+L) [polyclonal, HRP] JIR 111-035-144, 1:10,000 in TBST) for the detection of AIM lasted 2 h at RT. Detection was based on HRP with Clarity™ Western ECL substrate (Bio-Rad), followed by imaging with the ChemiDoc™ MP System (Bio-Rad). For secondary staining, the membrane was stripped with ROTI^®^Free Stripping-Buffer for 45 min at RT, preheated to 37°C, and washed prior to further ECL detection, re-blocking, and subsequent steps. As a protein ladder, ROTI^®^Mark TRICOLOR (Roth) was used. The following samples were analyzed for quality: recombinant AIM (R&D Systems), recombinant IgM (Polymun), human serum IgM (Merck), and Pentaglobin^®^ (Biotest).

**Results**

**Western Blot analysis**

Western blot analysis was performed to detect the expression of AIM in human serum IgM (Merck) and Pentaglobin^®^ (Biotest AG, Germany), compared to recombinant AIM (R&D Systems, USA) and recombinant IgM (Polymun, Austria). The analysis revealed that human serum and Pentaglobin^®^ contained AIM, as indicated by a distinct antibody detection of AIM at ~40 kDa (Supplementary Figure 1). In contrast, recombinant IgM did not display any detectable AIM signal.

**Prognostic Impact of free AIM on 30-day survival – ROC Analysis, AUC and multivariate Cox regression**

To further evaluate the prognostic value of free AIM levels in predicting 30-day survival, a Receiver Operating Characteristic (ROC) curve analysis was performed. The resulting ROC curves for free AIM levels at day 1, day 4, and day 8 are shown in Supplementary Figure 2. For day 1, the area under the curve (AUC) was 0.542 (CI: 0.420-0.665). The AUC values for day 4 and day 8 were 0.473 (CI: 0.341-0.606) and 0.655 (CI: 0.504-0.806), respectively.

The optimal cutoff values were derived by maximizing the Youden index and were then applied to stratify patients in the Kaplan Meier analysis shown in Figure 2. The corresponding metrics at these cutoffs were: day 1, sensitivity 0.091 and 1 − specificity 0.022 with a Youden index of 0.069; day 4, sensitivity 0.147 and 1 − specificity 0.048 with a Youden index of 0.099; and day 8, sensitivity 0.429 and 1 − specificity 0.156 with a Youden index of 0.272.

Furthermore a multivariate Cox regression was performed with the factors free AIM day 1, free AIM day 4, free AIM day 8, comorbidities and age and it was found that free AIM on day 8 [HR: 0.999 (0.999-1.000); p = 0.007], as well as cardiovascular comorbidity [HR: 0.242 (0.083-0.702); p = 0.009] and age [HR: 1.065 (1.021-1.110); p = 0.003] could be classified as independent risk factors for 30-day survival (see Supplementary Table 1).

**Prognostic Impact of total AIM on 30-day survival – ROC Analysis, AUC and multivariate Cox regression**

To examine the prognostic contribution of total AIM to 30-day survival, ROC curves were generated for days 1, 4, and 8 and are displayed in Supplementary Figure 3. The corresponding AUCs were 0.651 (CI: 0.538-0.764) for day 1, 0.622 (CI: 0.474-0.734) for day 4, and 0.608 (CI: 0.445-0.770) for day 8. Cutoff selection was based on the Youden-Index and these thresholds were then used to stratify patients in the Kaplan-Meier analysis shown in Figure 3. At the selected cutoffs, sensitivity and 1 - specificity were 0.891 and 0.636 on day 1, 0.441 and 0.163 on day 4, and 0.381 and 0.121 on day 8, with Youden-Index values of 0.255, 0.278, and 0.260, respectively. To test whether total AIM is an independent risk factor for 30-day survival a multivariate Cox regression was performed with the factors total AIM day 1, total AIM day 4, total AIM day 8, comorbidities and age and it was found that total AIM on day 1 [HR: 0.974 (0.955-0.993); p = 0.008], as well as cardiovascular comorbidity [HR: 0.205 (0.070-0.598); p = 0.004] and age [HR: 1.044 (1.004-1.086); p = 0.031] could be classified as independent risk factors for 30-day survival (see Supplementary Table 2).

**Figures**

******

**Supplementary Figure 1**: Western blot analysis was used to assess the expression of AIM in human serum IgM (Merck) and Pentaglobin^®^ (Biotest), in comparison to recombinant AIM (R&D Systems) and recombinant IgM (Polymun). For detection of the human IgM heavy chain, the goat anti-Human IgM Secondary Antibody, #31415, HRP (Invitrogen) was used. The IgM heavy chain band was observed at approximately ~80 kDa. AIM detection was carried out using the primary antibody Anti-CD5L/CT-2 antibody #ab45406 (Abcam) and the secondary antibody goat anti-rabbit IgG (H+L) [polyclonal, HRP] JIR 111-035-144. AIM was detected at approximately ~40 kDa. Both human serum IgM and Pentaglobin^®^ exhibited the characteristic signal for both the IgM heavy chain and AIM, while recombinant proteins only showed the corresponding band for each protein.


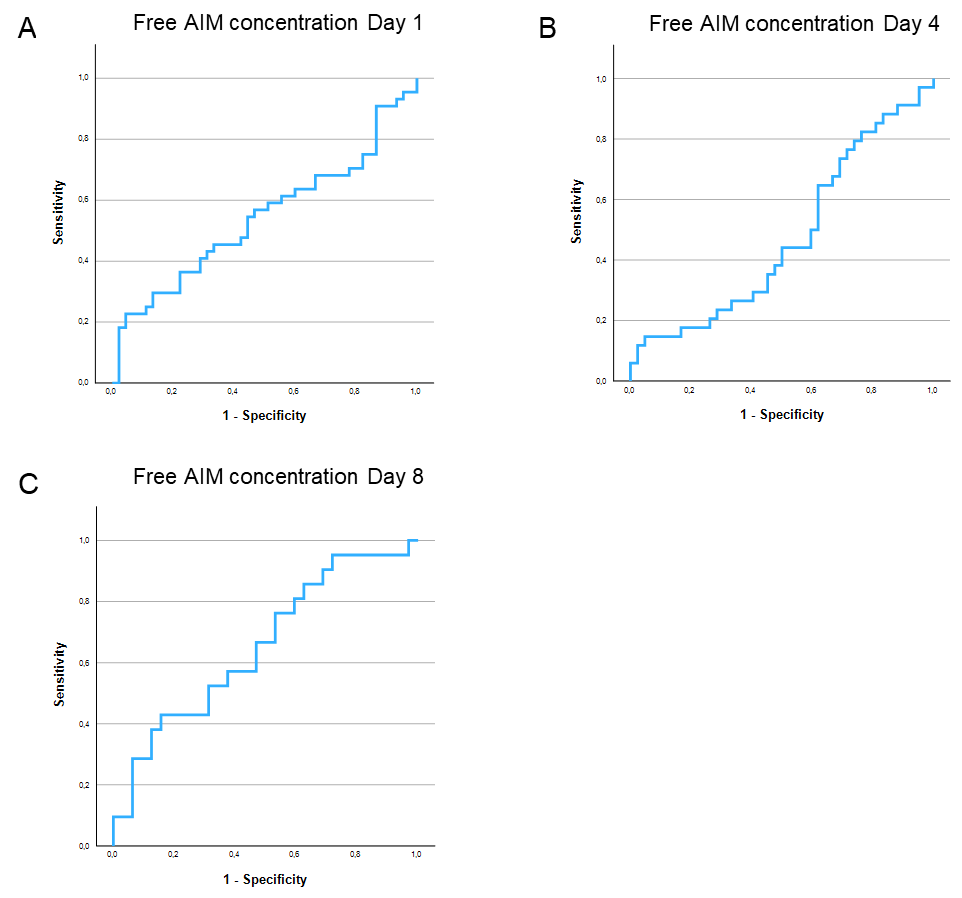


**Supplementary Figure 2:** ROC curves for free AIM concentrations. A. Free AIM concentration on day 1, AUC = 0.542 (CI: 0.420-0.665), sensitivity = 0.091, 1 – specificity = 0.022, Youden index = 0.069. B. Free AIM concentration on day 4, AUC = 0.473 (CI: 0.341-0.606), sensitivity = 0.147, 1 – specificity = 0.048, Youden index = 0.099. C. Free AIM concentration on day 8, AUC = 0.655 (CI: 0.504-0.806), sensitivity = 0.429, 1 – specificity = 0.156, Youden index = 0.272.

B

C

A

Total AIM concentration Day 1

Total AIM concentration Day 4

Total AIM concentration Day 8

**Supplementary Figure 3.** ROC curves for total AIM concentrations in relation to 30-day survival. A. Total AIM on day 1, AUC 0.651 (CI: 0.538-0.764), sensitivity 0.891, 1 − specificity 0.636, Youden-Index 0.255. B. Total AIM on day 4, AUC 0.622 (CI: 0.474-0.734), sensitivity 0.441, 1 − specificity 0.163, Youden-Index 0.278. C. Total AIM on day 8, AUC 0.608 (CI: 0.445-0.770), sensitivity 0.381, 1 − specificity 0.121, Youden-Index 0.260.

**Supplementary Table 1** **multivariate COX regression for free AIM.** A multivariate Cox regression was performed with the factors free AIM day 1, free AIM day 4, free AIM day 8, comorbidities and age and it was found that free-AIM on day 8 [HR: 0.999 (0.999-1.000); p = 0.007], as well as cardiovascular comorbidity [HR: 0.242 (0.083-0.702); p = 0.009] and age [HR: 1.065 (1.021-1.110); p = 0.003] could be classified as independent risk factors for 30-day survival.

| **Factor** | **Hazard ratio** | **95.0% CI** | **p-value** |
| --- | --- | --- | --- |
| Free AIM Day 1 | 1.000 | 0.999-1.000 | 0.429 |
| Free AIM Day 4 | 1.000 | 1.000-1.001 | 0.273 |
| **Free AIM Day 8** | 0.999 | 0.999-1.000 | **0.007** |
| Alcohol | 0.148 | 0.010-2.133 | 0.161 |
| Lung | 0.397 | 0.047-3.354 | 0.396 |
| Hypertension | 2.719 | 1.066-6.937 | 0.036 |
| CKP | 0.345 | 0.030-3.972 | 0.394 |
| COPD | 2.082 | 0.345-12.583 | 0.424 |
| Diabetes | 1.767 | 0.517-6.037 | 0.364 |
| Obesity | 1.127 | 0.384-3.307 | 0.827 |
| **Cardiovascular** | 0.242 | 0.083-0.702 | **0.009** |
| Malignant | 1.649 | 0.589-4.618 | 0.341 |
| Nicotine | 2.422 | 0.281-20.849 | 0.421 |
| **Age** | 1.065 | 1.021-1.110 | **0.003** |

**Supplementary Table 2 multivariate COX regression for total AIM.** A multivariate Cox regression was performed with the factors total AIM day 1, total AIM day 4, total AIM day 8, comorbidities and age and it was found that total AIM on day 1 [HR: 0.974 (0.955-0.993); p = 0.008], as well as cardiovascular comorbidity [HR: 0.205 (0.070-0.598); p = 0.004] and age [HR: 1.044 (1.004-1.086); p = 0.031] could be classified as independent risk factors for 30-day survival.

| **Factor** | **Hazard ratio** | **95.0% CI** | **p-value** |
| --- | --- | --- | --- |
| **Total AIM Day 1** | 0.974 | 0.955-0.993 | **0.008** |
| Total AIM Day 4 | 1.009 | 0.997-1.021 | 0.129 |
| Total AIM Day 8 | 0.985 | 0.966-1.004 | 0.119 |
| Alcohol | 0.087 | 0.008-0.986 | 0.049 |
| Lung | 0.168 | 0.023-1.239 | 0.080 |
| Hypertension | 1.555 | 0.561-4.308 | 0.396 |
| CKP | 0.556 | 0.046-6.699 | 0.644 |
| COPD | 4.036 | 0.772-21.115 | 0.098 |
| Diabetes | 0.961 | 0.291-3.171 | 0.948 |
| Obesity | 1.284 | 0.444-3.716 | 0.644 |
| **Cardiovascular** | 0.205 | 0.070-0.598 | **0.004** |
| Malignant | 1.018 | 0.349-2.968 | 0.974 |
| Nicotine | 0.697 | 0.113-4.310 | 0.698 |
| **Age** | 1.044 | 1.004-1.086 | **0.031** |
